# Characterization and Genomic Analysis of Novel *Vibrio parahaemolyticus* Phage vB_VpaP_DE10

**DOI:** 10.3390/v14081609

**Published:** 2022-07-23

**Authors:** Yuanming Ye, Hanfang Chen, Qiaolan Huang, Shixuan Huang, Jiaxin He, Jumei Zhang, Qingping Wu, Xueling Li, Wenfeng Hu, Meiyan Yang

**Affiliations:** 1 College of Agriculture, College of Food Science, South China Agricultural University, 483 Wushan Road, Guangzhou 510642, China; yeym0424@163.com (Y.Y.); chenhanfangh@163.com (H.C.); symxmyz3133@163.com (Q.H.); nhuhuganaxis@oulook.com (S.H.); hjxstar1217@163.com (J.H.); lixueling@scau.edu.cn (X.L.); 2 Guangdong Provincial Key Laboratory of Microbial Safety and Health, State Key Laboratory of Applied Microbiology Southern China, Institute of Microbiology, Guangdong Academy of Sciences, Guangzhou 510070, China; zhangjm926@126.com (J.Z.); wuqp203@163.com (Q.W.)

**Keywords:** *Vibrio parahaemolyticus*, phage, *Autographiviridae*, genome analysis

## Abstract

In the present study, a novel lytic *Vibrio parahaemolyticus* phage, vB_VpaP_DE10, was isolated from sewage samples collected in Guangzhou city, China. Transmission electron microscopy revealed that phage vB_VpaP_DE10 has an icosahedral head (52.4 ± 2.5 nm) and a short non-contracted tail (21.9 ± 1.0 nm). Phage vB_VpaP_DE10 lysed approximately 31% (8/26) of the antibiotic-resistant *V. parahaemolyticus* strains tested. A one-step growth curve showed that phage vB_VpaP_DE10 has a relatively long latency time of 25 min and a burst size of ~19 PFU per cell. The genome of phage vB_VpaP_DE10 is a 42,871-bp-long dsDNA molecule with a G + C content of 49.19% and is predicted to contain 46 open reading frames, 26 of which are predicted to be related to functions such as phage structure, packaging, host lysis, and DNA metabolism. Sequence comparisons suggested that vB_VpaP_DE10 is a member of the genus *Maculvirus* within the family *Autographiviridae*. Morphological and genomic analysis indicated that vB_VpaP_DE10 is a novel *V. parahaemolyticus* phage.

## 1. Introduction

*Vibrio parahaemolyticus* is a Gram-negative bacterium that exists in marine, riverine, and aquaculture environments [1]. Infection by this bacterium can lead to gastroenteritis, fever, and sepsis [2]. *V. parahaemolyticus* was first discovered in Osaka, Japan, and has been prevalent ever since [3]. According to the U.S. Centers for Disease Control and Prevention (CDC), approximately 6680 cases of *V. parahaemolyticus* infection are reported each year with an estimated annual health cost of more than 30 million dollars [4]. In China, *V. parahaemolyticus* has been the main cause of foodborne disease outbreaks and infectious diarrhea cases in coastal areas [5]. From 2013 to 2017, a total of 1220 strains of *V. parahaemolyticus* were isolated from 16,504 fecal specimens of patients with acute diarrhea in southeastern China; the annual isolation rate fluctuated between 6.1 and 8.7% [6]. The pathogenic factors of *V. parahaemolyticus* are mainly related to two virulence factors, namely thermostable direct hemolysin (TDH) and TDH-related hemolysin (TRH) [7]. Both toxins have hemolytic and enterotoxic activities and are cardiotoxic and cytotoxic [8].

The use of antibiotics is a common practice in treating the disease caused by infection with this bacterium; however, over time, an increasing number of reports have shown that *V. parahaemolyticus* isolated from various sources is highly resistant to single or multiple antibiotics, particularly ampicillin [9]. Disinfectants are also traditional countermeasures to control *V. parahaemolyticus*, but they are non-specific, easily destroy the natural ecology of aquaculture water, and the resulting residues pose a threat to the health of consumers [10]. Therefore, environmentally friendly methods are needed to replace traditional measures to control *V. parahaemolyticus*.

In the past decade, studies have suggested that phage therapy may be a promising strategy to control *V. parahaemolyticus* [11]. However, at present, there are very limited phage resources. As of 22 April 2022, there were 24,646 phage strains recorded in NCBI, including 24,426 culturable strains, and only 78 *V. parahaemolyticus* phage strains in total. Therefore, obtaining sufficient phage resources is a top priority. Moreover, it is necessary to clear the genetic information of phages before application. Considering the high genetic diversity and mutation rate of phages, traditional or single microbial analysis methods are no longer applicable. Therefore, it is very important to continuously update research methods and use multiple methods to verify each other.

In this study, we isolated a *V. parahaemolyticus* phage, vB_VpaP_DE10, from the sewage of the Huangsha Aquatic Product Market in Guangzhou, China. We assessed the basic biological properties of its lytic activity, namely host range and one-step growth curve, and performed a multi-angle comprehensive analysis of its whole genome.

## 2. Materials and Methods

### 2.1. Bacterial Strains and Growth Conditions

Twenty-six *V. parahaemolyticus* strains provided by the Institute of Microbiology, Guangdong Academy of Sciences, were used in this study. The serotype, resistance, and origin of each strain were confirmed by previous studies [12] (Table 1). *V. parahaemolyticus* O4-12 was used as a host for phage isolation and propagation. Strains were stored at −80 °C in 30% (*v*/*v*) glycerol. All *V. parahaemolyticus* strains were cultured in 5 mL of tryptic soy broth (TSB) and incubated at 37 °C for 12 h with shaking.

### 2.2. Isolation of Phage

Phage vB_VpaP_DE10 was isolated from sewage samples collected in the Huangsha aquatic product market in Guangzhou city, China, by a previously described method [13]. Sewage samples were subjected to centrifugation to remove large particulates (5000× *g* for 10 min), and the supernatant was filtered using a 0.45 μm filter (HuanKai Microbial, Guangzhou, China). Thereafter, MgSO_4_ was added to the filtrate to a final concentration of 50 mM and allowed to stand for 10 min before filtering using a 0.22-μm filter (HuanKai Microbial, Guangzhou, China). The filter membranes were cut and eluted with a broth containing 3% (*w*/*v*) Bacto beef extract, 3% (*v*/*v*) Tween 80, and 50 mM NaCl. Subsequently, 2 mL of eluent was mixed with 2 mL of sterile double-strength TSB (4 mM CaCl_2_) and an 80-µL *V. parahaemolyticus* O4-12 culture (OD_600_ = 0.2). The mixtures were incubated overnight at 37 °C with shaking. After incubation, the mixed cultures were centrifuged at 8000× *g* for 5 min, and the supernatant was filtered through a 0.45 μm syringe filter. The purified phages were incubated with *V. parahaemolyticus* again at least twice to enrich the phage particles. A spot test assay was performed to test for the presence of lytic phages. The double-layer agar method was used to obtain single plaques and a plaque was purified four times to ensure the isolated phage was pure [14].

### 2.3. Phage Concentration and Morphological Observation

Phage concentration was performed as previously described, with modifications [15]. First, 0.1 mL phage lysate sediment was incubated in 50 mL TSB at 37 °C for 4 h, then centrifuged at 5000× *g* for 10 min and filtered through a 0.45-μm-pore membrane. Thereafter, polyethylene glycol (15% *w*/*v* PEG8000, 0.5 M NaCl) was added to it and placed at 4 °C overnight.

The overnight phage concentrate was centrifuged at 12,000× *g* for 20 min at 4 °C. The sediment was resuspended in SM buffer. The CsCl density gradient centrifugation method was used to purify and concentrate phages [16]. Phage particles located near the 1.5-g/mL density band were collected and their morphological features were observed under a transmission electron microscope (Hitachi H-7650; Hitachi, Tokyo, Japan).

### 2.4. Spot Assay

First, 100 μL of bacterial culture in the mid-log phase was mixed with 5 mL TSB (0.4% agar and 2 mM CaCl_2_), poured onto tryptic soy agar plates, and placed at room temperature for 5 min or until the agar solidified. Then, 2 µL of tenfold diluted phage stock were pipetted on each plate. Thereafter, the plates were incubated at 37 °C for 6 h. Based on the clarity of plaques, the results were divided into two categories: clear (+) and no plaques (-) in the spotting area.

### 2.5. Efficiency of Plating (EOP)

All the *V. parahaemolyticus* isolates which were sensitive to the phage vB_VpaP_DE10 in the spot test were selected for the determination of EOP by the double-agar layer method [17]. Briefly, tenfold serial dilutions of phage suspensions (100 μL) were added to 5 mL TSB-soft agar with 100 μL *V. parahaemolyticus* culture (10^8^ CFU/mL). The mixture was poured onto TSA plates and incubated at 37 °C for 6 h. The EOP values were calculated as the ratio of lysis plaques produced in each susceptible strain divided by the number of the plaques produced in *V. parahaemolyticus* O4-12 and then were ranked as “high efficiency” (EOP ≥ 0.5), “medium efficiency” (0.1 ≤ EOP < 0.5), “low efficiency” (0.001 < EOP < 0.1), or “inefficient” (EOP ≤ 0.001).

### 2.6. Phage Nucleotide Extraction

Phage whole-genome extraction was performed as described by Xing S et al. [18]. The phage concentrate was centrifuged at 12,000× *g* for 20 min at 4 °C, and the supernatant was discarded. Subsequently, 1 mL SM buffer was added to fully wash the tube wall and precipitate DNA, following which DNase I and RNase A were added to final concentrations of 0.1 units/µL and 3 µg/mL, respectively. The mixture was incubated at 37 °C for 1 h. Next, purified phages were treated with 10% SDS, EDTA, and proteinase K for 30 min at 65 °C. Then, an equal volume of Tris-saturated phenol solution was added and the mixture was centrifuged at 12,000× *g* for 5 min. The upper aqueous phase was transferred to another centrifuge tube, an equal volume of premixed phenol-chloroform-isoamyl alcohol (25:24:1) was added to it, and the mixture was centrifuged at 12,000× *g* for 5 min. The upper aqueous phase was again transferred to a fresh centrifuge tube, and an equal volume of chloroform was added (this step was repeated thrice). Thereafter, the upper aqueous phase was pipetted into a fresh centrifuge tube, an equal volume of isopropanol was added to it, and the tube was then incubated at −20 °C for 30 min. Next, the DNA sediment was collected by centrifugation at 12,000× *g* for 5 min at 4 °C, washed with 70% ethanol, and centrifuged at 12,000× *g* for 5 min again (this step was repeated twice). Subsequently, the DNA sediment was dried at room temperature and 50 μL deionized water (preheated to 65 °C) was added to it. The extracted DNA was stored at −20 °C.

### 2.7. Genome Sequencing and Bioinformatics Analysis

The phage DNA was sequenced using Ion Torrent S5 platform (Thermo Fisher Scientific, Waltham, MA, USA). High-quality reads were assembled using SPAdes v. 3.12.0 [19]. The whole-genome sequence was aligned with phage sequences in GenBank using BLASTN and then analyzed by average nucleotide identity (ANI). Genome annotation was conducted using Prokka [20]. Putative tRNA-encoding genes were predicted using tRNAscan-SE (http://lowelab.ucsc.edu/tRNAscan-SE/ accessed on 18 November 2021). The virulence factors and antibiotic genes were predicted using the virulence factor database [21] (VFDB, http://www.mgc.ac.cn/VFs/ accessed on 18 November 2021) and antibiotic resistance gene database [22] (ARDB, http://ardb.cbcb.umd.edu/ accessed on 18 November 2021), respectively. A circular map of the phage vB_VpaP_DE10 genome was generated using CGView [23]. For phylogenetic analysis, the amino acid sequences of DNA polymerase and RNA polymerase were selected for multiple alignments, firstly using the Clustal W algorithm [24]. Based on the alignments, a neighbor-joining (NJ) phylogenetic tree was constructed in MEGA-X using the Jones–Taylor–Thornton model [25,26]. Bootstrap values were calculated from 1000 replicates [27]. The Newick format was used to create the phylogenetic tree by iTOL (https://itol.embl.de accessed on 18 June 2022). Finally, the whole phage genome was visualized using Easyfig2.2.3 [28].

### 2.8. Phage One-Step Growth Curve Assay

The one-step growth curve of phage vB_VpaP_DE10 was tested according to a previous method with minor modifications [15]. Briefly, 1 mL of the diluted bacterial culture (1 × 10^8^ CFU/mL) was centrifuged at 8000× *g* for 5 min, the sediment was resuspended in 1 mL SM buffer, and 100 µL phage at multiplicity of infection (MOI) = 0.1 was added to it. This mixture was incubated at 37 °C for 10 min and the mixture was then centrifuged and resuspended in 1 mL TSB. Next, 0.2 mL of this mixture was added to 19.8 mL of TSB and incubated at 37 °C and 200 r/min for 50 min. Samples were taken every 5 min to determine the phage titer from 0 min. The experiment was repeated thrice. The burst size was calculated as follows:

$$\mathrm{burst}\;\mathrm{size}=\frac{\mathrm{average}\;\mathrm{phages}\;\mathrm{of}\;\mathrm{the}\;\mathrm{plateau}\;\mathrm{period}}{\mathrm{amount}\;\mathrm{of}\;\mathrm{infective}\;\mathrm{bacterial}\;\mathrm{cells}}$$

### 2.9. Accession Number

The whole-genome sequence of phage vB_VpaP_DE10 was deposited in GenBank under the accession number MZ516827.

### 2.10. Statistical Analysis

The data were expressed as mean ± standard deviation (SD) and the differences were analyzed with two-way ANOVA using GraphPad Prism 8.4.2. Significance was considered at *p* < 0.05.

## 3. Results

### 3.1. Isolation, Identification, and General Characterization of Phages

**Figure 1:**
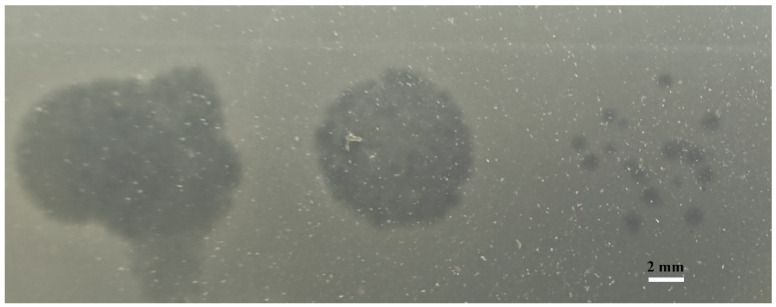
The serial dilution spot test of phage vB_VpaP_DE10 against *V. parahaemolyticus* O4-12.

**Figure 2:**
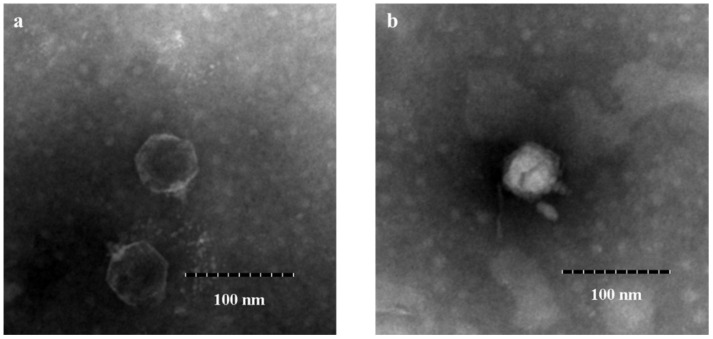
Morphology of phage vB_VpaP_DE10 observed using transmission electron microscopy (**a**) after DNA injection and (**b**) without DNA injection.

**Figure 3:**
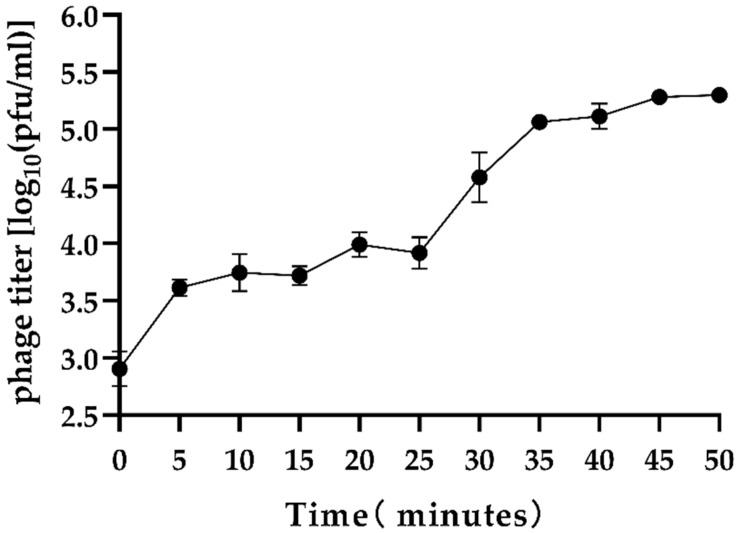
One-step growth curve of phage vB_VpaP_DE10.

The phage vB_VpaP_DE10 was isolated from sewage using antibiotic-resistant *V. parahaemolyticus* O4-12 as the host cell. It was able to form clear plaques approximately 1–2 mm in diameter (Figure 1). Transmission electron microscopy (Figure 2) revealed that phage vB_VpaP_DE10 has an icosahedral head of 52.4 ± 2.5 nm and a short non-contracted tail of 21.9 ± 1.0 nm. Based on the one-step growth curves shown in Figure 3, phage vB_VpaP_DE10 was characterized by a latency period of 0–25 min, a lysis period of 25–35 min, and a burst size of approximately 19 PFU per cell.

### 3.2. Host Range

A total of 26 strains of antibiotic-resistant *V. parahaemolyticus*, which belonged to 11 different kinds of O antigens, were used to evaluate the host range of phage vB_VpaP_DE10. In spot tests, phage vB_VpaP_DE10 formed clear spots on the lawns of 10 out of 26 *V. parahaemolyticus* strains (Table 1), indicating that these strains were sensitive to the phage suspensions. Thus, these 10 *V. parahaemolyticus* strains were used for the EOP determination. Results showed that vB_VpaP_DE10 only formed plaques on 8 *V. parahaemolyticus* strains by the double-layer agar plate method but did not form plaques on *V. parahaemolyticus* O6-20 and O8-21, indicating that though it inhibited the growth of *V. parahaemolyticus* O6-20 and O8-21, it did not lyse them.

### 3.3. Genomic Signature of Phage vB_VpaP_DE10

**Figure 4:**
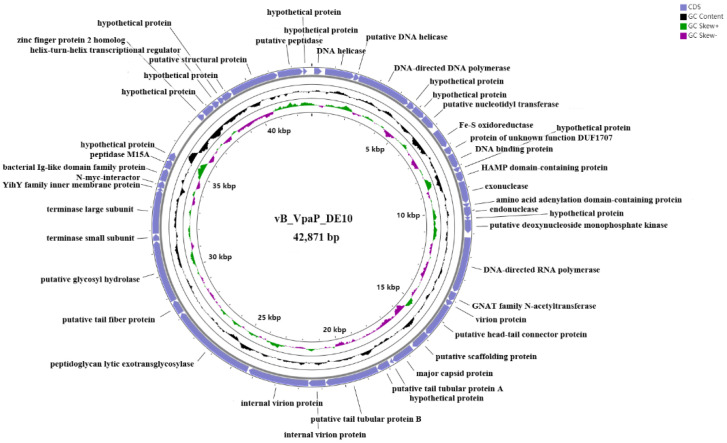
*Vibrio parahaemolyticus* phage vB_VpaP_DE10 genome. Circles display (from the outside) the following: (1) Open reading frames transcribed in the clockwise or the counterclockwise direction. (2) G + C % content. Values > 49.19 % (average) are shown as outward peaks, and smaller values are shown as inward peaks. (3) GC skew. (4) Physical map scaled in kbp.

**Figure 5:**
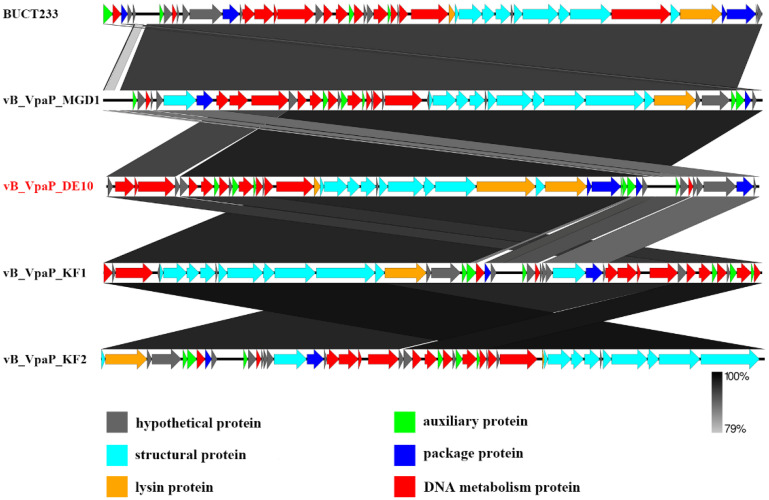
Genome comparison of vB_VpaP_DE10, BUCT233, vB_VpaP_MGD1, and vB_VpaP_KF1–2 phages using the Easyfig tool. Different colored arrows represent 46 predicted open reading frames with different functions.

To better understand the phage vB_VpaP_DE10, its genomic DNA was extracted and sequenced. As shown in Figure 4, the phage vB_VpaP_DE10 genome consisted of 42,871 bp of double-stranded DNA with an average G + C content of 49.19% and 46 open reading frames (ORFs) with an average length of 861 bp and varying in size from 93 to 3855 bp. A total of 39,599 nucleotides (accounting for 92.37% of the total genome) formed the coding sequence. As shown in Figure 5 and Table 2, among the annotated 46 ORFs, 26 were predicted as functional proteins, including ten phage DNA metabolism-related proteins (ORF2, ORF3, ORF4, ORF7, ORF10, ORF13, ORF15, ORF17, ORF18, and ORF19), ten morphogenesis-related proteins (ORF20, ORF21, ORF22, ORF23, ORF25, ORF26, ORF27, ORF28, ORF30, and ORF44), four lysis-related functional proteins (ORF29, ORF31, ORF37, and ORF45), and two packaging-related proteins (ORF32 and ORF33).The presence of the RNA polymerase gene suggested that vB_VpaP_DE10 may belong to the described family *Autographiviridae*. *Autographivirinae* was once considered a subfamily of the *Podoviridae* family whose defining feature was the presence of virally encoded RNA polymerases [29]. Further common characteristics of these phages include conservation of gene arrangement and apparently genus-specific lysis cassettes and RNAP specificity loops. In 2019, ICTV removed the *Autographivirinae* and *Autographivirinae*-like viruses from the family *Podoviridae* and assigned a family rank, “*Autographiviridae*” [30]. In addition, no tRNA-encoding, antibiotic resistance, lysogenic, or virulence genes were observed upon genomic analysis.

**Figure 6:**
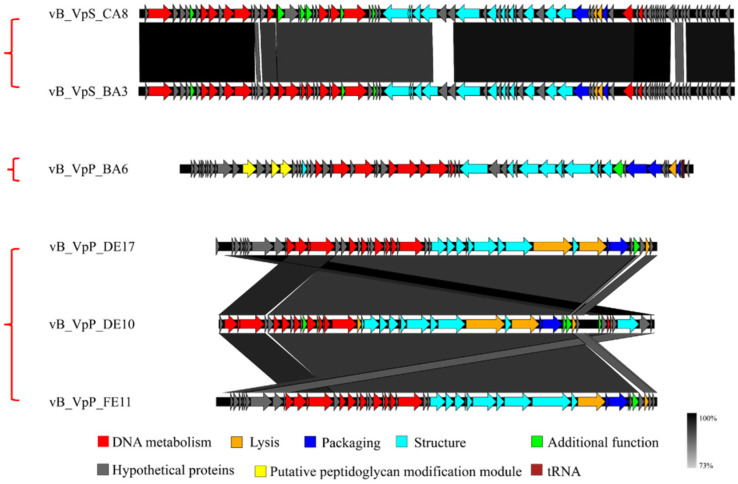
Genome comparison of vB_VpaP_DE10, vB_VpP_BA6, vB_VpS_BA3, vB_VpS_CA8, and vB_VpP_DE17, using Easyfig.

In order to investigate the genetic diversities of *Vibrio* phages isolated by our group, the whole genome alignment between phage vB_VpaP_DE10 and phages vB_VpP_BA6 [13], vB_VpP_DE17 [15], vB_VpP_FE11 [16], vB_VpS_BA3, and vB_VpS_CA8 [12] was performed. As showed in Figure 6, sequence comparison allowed for clear discrimination of three kinds of genomes. Genomes of *Siphoviridae* phages vB_VpS_BA3 and vB_VpS_CA8 represented type 1, the genome of *Podoviridae* phage vB_VpS_BA6 represented type 2, and genomes of *Autographiviridae* phage vB_VpaP_DE10, vB_VpP_DE17, and vB_VpP_FE11 all represented type 3.

### 3.4. Phage Taxonomy and Phylogeny Analysis

In order to know the genome profile of phage vB_VpaP_DE10, the whole-genome BLASTn analysis was performed first. The genome of vB_VpaP_DE10 showed a high homology (>95%) with genomes of eight distinct species of the genus *Maculvirus* (Table 3). Among them, the highest sequence similarity was given by the phage vB_VpaP_MGD1 (97.02% identity, 86% query coverage). When comparing the 46 ORFs of vB_VpaP_DE10 with vB_VpaP_MGD1, 32 showed ≥95% identity, 4 showed 85% to 95% identity, 3 showed <85% identity; and 7 showed no homology at all. In the 7 ORFs with no homology, 4 were annotated as hypothetical proteins (ORF 1, 5, 42, 46); the others were annotated as DNA helicase (ORF 2), YihY family inner membrane protein (ORF 19), and GNAT family N-acetyltransferase (ORF 34), respectively. They were all present in vB_VpaP_DE10 and absent in vB_VpaP_MGD1.

**Figure 7:**
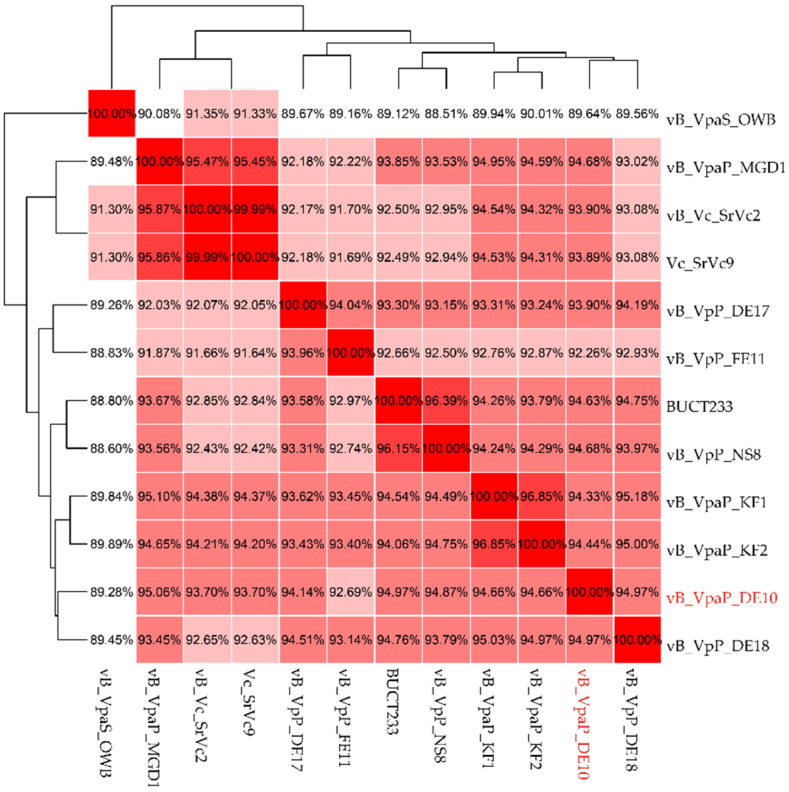
Heatmap of the ANI values for 12 whole genomes of the family *Autographiviridae*. Values range from 0 (0% ANI) to 1 (100% ANI): clusters of highly similar phages are highlighted in pink and red.

Due to the high genetic diversity of viruses, ANI analysis was suggested for their taxonomy in recent years. Thus, in order to accurately classify the phage vB_VpaP_DE10, ANI analysis between *Vibrio* phages of the genus *Maculvirus* was performed. As shown in Figure 7, the ANI values of vB_VpaP_DE10 with the 11 phages which had the highest BLASTN similarities were between 89.46% and 94.97% (Figure 7). As the threshold of ANI values to distinguish the viral species was below 95%, phage vB_VpaP_DE10 was considered as a new phage species [30,31].

**Figure 8:**
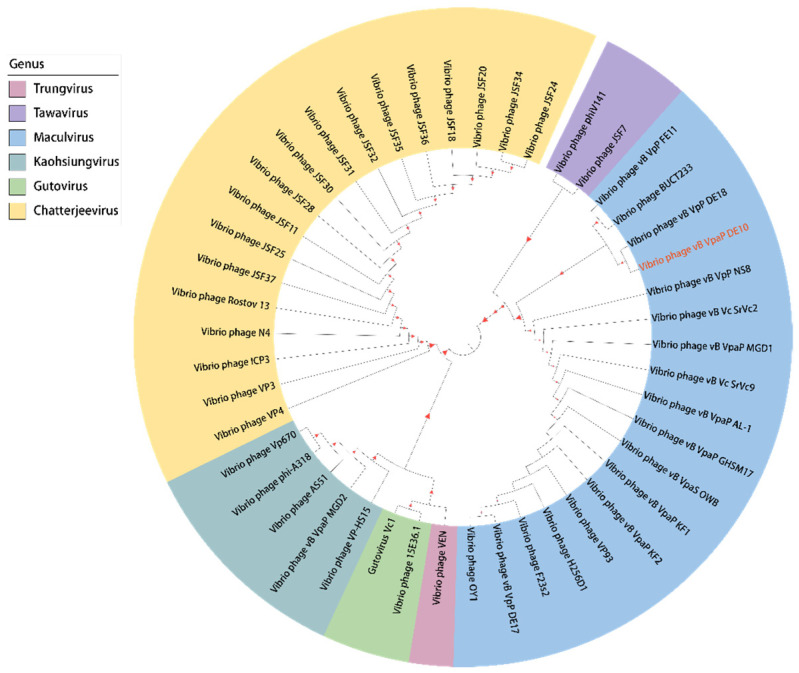
Phylogenetic tree based on the DNA polymerase.

**Figure 9:**
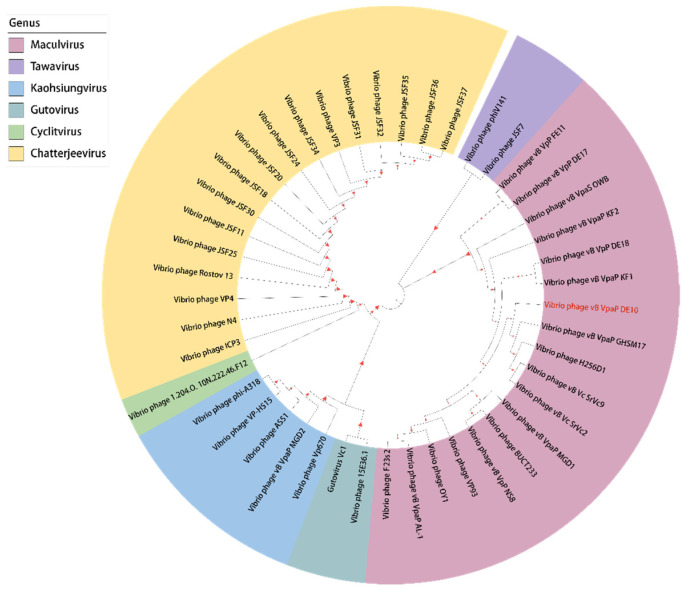
Phylogenetic tree based on the RNA polymerase.

For phylogenetic analysis of phage vB_VpaP_DE10, the DNA polymerase and RNA polymerase were analyzed with all the *Vibrio* phages of the family *Autographiviridae* family. In the DNA polymerase-based phylogenetic tree, phage vB_VpaP_DE10 was most closely related to the phage vB_VpP_DE18 of the genus *Maculvirus* (Figure 8). When in the RNA polymerase-based tree, vB_VpaP_DE10 was also clustered with four *Maculvirus* phages (vB_VpaP_GHSM17, H256D1, vB_Vc_SrVc2, and vB_Vc_SrVc9 (Figure 9). Therefore, phage vB_VpaP_DE10 was considered as a member of the genus *Maculvirus*, which belongs to the family *Autographiviridae*.

## 4. Discussion

In this study, phage vB_VpaP_DE10 was obtained from sewage in the aquatic market, from where several *Vibrio* phages were isolated by our group at a different time. Although originating from the same place, genomes of six *Vibrio* phages showed high genetic diversities, and taxonomy varied. For example, even within the group of short-tail phages, the genome of vB_VpP_BA6 was completely different from vB_VpP_DE17, vB_VpP_FE11, and vB_VpaP_DE10.

Based on the virionic morphology and genome analysis, phage vB_VpaP_DE10 was a member of the *Autographiviridae* family. Several *Autographiviridae* phages specific for *V. parahaemolyticus* have been studied, such as phage vB_VpP_DE17 [15], phage vB_VpP_FE11 [16], phage vB_VpaP_MGD2 [14], phage vB_VpaP_AL-1 [32], phage VP93 [33], phage OWB [34], phage vB_VpaP_KF1, and phage vB_VpaP_KF2 [35]. Each phage has a different growth profile that can be used to evaluate the potential for application. Previous studies on *V. parahaemolyticus* phages of *Autographiviridae* family showed a latency period of 5–30 min and burst size of 37–244 PFU per cell [14,15,16,32]. Compared with these reports, vB_VpaP_DE10 had a relatively long latency period and a small burst size.

In addition, the phage was also tested for the ability to lyse other bacteria for assessment of host range [17,36]. Both spot assay and EOP test of phage vB_VpaP_DE10 were performed. Results showed that the EOP values varied widely among the 10 antibiotic-resistant strains. Values of *V. parahaemolyticus* O3-11 and O11-31 were higher than the indicated host *V. parahaemolyticus* O4-12, whereas others were lower. Changes of surface molecules that serve as phage receptors would significantly affect EOP and reduce phage adsorption [37,38]. Therefore, EOP differences are likely the result of differences in bacterial receptors.

For the host range difference, phages vB_VpaP_DE10 and vB_VpaP_FE11 both belong to the *Maculvirus* genus of the family *Autographiviridae*, and have short tails and high sequence similarity; however, the phage vB_VpaP_FE11 could infect *V. parahaemolyticus* O6-20, O8-21, and O11–30 strains whereas vB_VpaP_DE10 could not. The host range of phages has been reported to be associated with tail fiber or receptor-binding proteins [39]. Mutations of tail-related proteins could alter the host range of phages [40]. In the structure module of the vB_VpaP_DE10 genome, three tail-related proteins were predicted, including the tail tubular protein A/B (TTPA/TTPB, Gp25/26) and tail fiber protein (Gp30). These three proteins were also predicted in the genome of phage FE11, and their amino acid sequences have high similarity to that of phage of vB_VpaP_DE10 (96.77%, 95.00%, and 96.55%, respectively). The reason why the host ranges of the two phages differ may be due to the differences in their amino acid residues of 3–5%. Further studies are required to confirm this hypothesis. In conclusion, we isolated and characterized a novel lytic *V. parahaemolyticus* phage, vB_VpaP_DE10, having a short tail, and belonging to the genus *Maculvirus* within the family *Autographiviridae*.

## Figures and Tables

**Table 1:** Antibiotic resistance profile of *V. parahaemolyticus* strains and host range of phage vB_VpaP_DE10.

No.	*V. parahaemolyticus* Strains	Serotype	Antibiotic Resistance	Spot Test	EOP Value	EOP Rank
1	O1-1	O1	CN-K-CIP-S-AMP	-	NT	Inefficient
2	O1-3	O1	K-CIP-S-AMP-C	+	0.32	Medium
3	O2-5	O2	S-AMP	-	NT	Inefficient
4	O2-7	O2	CIP-S-AMP	-	NT	Inefficient
5	O3-8	O2	K-CIP-S-AMP	-	NT	Inefficient
6	O3-10	O3	AMP	-	NT	Inefficient
7	O3-11	O3	CN-S-AMP-C	-	2.80	High
8	O4-12	O4	CIP-S-AMP	+	1	High
9	O4-13	O4	S-AMP	+	0.99	High
10	O4-14	O4	AMP	+	0.40	Medium
11	O5-15	O5	CN-K-CIP-S-AMP	+	NT	Inefficient
12	O6-18	O6	K-CIP-S-AMP	+	0	Inefficient
13	O6-20	O6	CIP-S-AMP	+	0.68	High
14	O8-21	O8	S-AMP	+	0	Inefficient
15	O8-126	O8	AMP	-	NT	Inefficient
16	O9-24	O9	CN-CIP-S-AMP	-	NT	Inefficient
17	O10-25	O10	S-AMP	-	NT	Inefficient
18	O10-26	O10	CN-CIP-S-AMP-C	-	NT	Inefficient
19	O10-28	O10	AMP	-	NT	Inefficient
20	O11-29	O11	CN-K-CIP-AMP	-	NT	Inefficient
21	O11-30	O11	S-AMP	-	NT	Inefficient
22	O11-31	O11	CIP-S-AMP	+	8.91	High
23	O12-32	O12	S-AMP	-	NT	Inefficient
24	O12-33	O12	CIP-S-AMP	+	0.065	Low
25	O12-34	O12	SXT-CIP-AMP-TE-K	-	NT	Inefficient
26	O12-35	O12	CN-CIP-S-AMP	-	NT	Inefficient

CN: gentamicin, K: kanamycin, CIP: ciprofloxacin, S: streptomycin, AMP: ampicillin, C: chloramphenicol, SXT: sulfamethoxazole-trimethoprim, TE: tetracycline. Clear plaque (+); no plaque (-).

**Table 2:** Predicted ORFs of *V. parahaemolyticus* phage vB_VpaP_DE10.

Label	Length (nt|aa)	Product	Organism	Identity
ORF2	1281|426	DNA helicase	*Vibrio* phage vB_VpP_DE18	100%
ORF3	234|77	putative DNA helicase	*Vibrio* phage vB_VpP_DE18	100%
ORF4	2427|808	DNA-directed DNA polymerase	*Vibrio* phage vB_VpP_DE18	99%
ORF7	597|198	putative nucleotidyl transferase	*Vibrio* phage vB_VpaP_KF1	98%
ORF10	630|209	DNA binding protein	*Vibrio* phage BUCT233	98%
ORF13	951|316	exonuclease	*Vibrio* phage vB_VpP_DE18	99%
ORF15	441|146	endonuclease	*Vibrio* phage BUCT233	100%
ORF17	585|194	putative deoxynucleoside monophosphate kinase	*Vibrio* phage vB_VpaP_KF1	80%
ORF18	2451|816	DNA-directed RNA polymerase	*Vibrio* phage BUCT233	99%
ORF19	420|139	GNAT family N-acetyltransferase	*Vibrio* phage vB_VpP_DE18	100%
ORF20	246|81	virion protein	*Vibrio* phage vB_VpP_DE18	100%
ORF21	1533|510	putative head–tail connector protein	*Vibrio* phage vB_VpP_DE17	99%
ORF22	816|271	putative scaffolding protein	*Vibrio* phage vB_VpaP_MGD1	99%
ORF23	999|332	major capsid protein	*Vibrio* phage vB_VpaP_MGD1	99%
ORF25	561|186	putative tail tubular protein A	*Vibrio* phage vB_VpaP_MGD1	100%
ORF26	2343|780	putative tail tubular protein B	*Vibrio* phage vB_VpaP_MGD1	99%
ORF27	741|246	internal virion protein	*Vibrio* phage vB_VpP_FE11	86%
ORF28	2679|892	internal virion protein	*Vibrio* phage vB_VpaP_KF1	95%
ORF29	3855|1284	peptidoglycan lytic exotransglycosylase	*Vibrio* phage vB_VpP_DE18	99%
ORF30	612|203	putative tail fiber protein	*Vibrio* phage VP93	99%
ORF31	2733|910	putative glycosyl hydrolase	*Vibrio* phage vB_VpaP_KF1	99%
ORF32	300|99	terminase small subunit	*Vibrio* phage vB_VpP_DE18	98%
ORF33	1920|639	terminase large subunit	*Vibrio* phage vB_Vc_SrVc2	100%
ORF37	414|137	peptidase M15A	*Vibrio* phage vB_VpP_DE18	100%
ORF44	2136|711	putative structural protein	*Vibrio* phage vB_VpP_DE18	98%
ORF45	1092|363	putative peptidase	*Vibrio* phage vB_VpP_DE18	100%

**Table 3:** Phages with high genome homology (>95%) to phage vB_VpaP_DE10.

Accession Number	Virus Name	Identity (%)
MT501516.1	vB_VpaP_MGD1	96.41
NC_048035.1	vB_VpaP_KF1	95.97
MZ592921.1	vB_VpP_NS8	95.87
MZ020222.1	BUCT233	95.64
MW331544.1	vB_Vc_SrVc2	95.55
LR794124.1	vB_Vc_SrVc9	95.55
NC_048036.1	vB_VpaP_KF2	95.21
MZ182247.1	vB_VpP_DE18	95.06

## Data Availability

The findings of this study are available within this paper. The complete genome sequence of phage vB_VpaP_DE10 was submitted to the GenBank database under accession number MZ516827.
